# Multiplex core of the human brain using structural, functional and metabolic connectivity derived from hybrid PET-MR imaging

**DOI:** 10.3389/fnimg.2023.1115965

**Published:** 2023-08-14

**Authors:** Martijn Devrome, Koen Van Laere, Michel Koole

**Affiliations:** ^1^Nuclear Medicine and Molecular Imaging, Department of Imaging and Pathology, Katholieke Universiteit (KU) Leuven, Leuven, Belgium; ^2^Division of Nuclear Medicine, Universitair Ziekenhuis (UZ) Leuven, Leuven, Belgium

**Keywords:** brain connectivity, PET-MR imaging, multilayer network, structural connectivity, functional connectivity, metabolic connectivity, aging

## Abstract

With the increasing success of mapping brain networks and availability of multiple MR- and PET-based connectivity measures, the need for novel methodologies to unravel the structure and function of the brain at multiple spatial and temporal scales is emerging. Therefore, in this work, we used hybrid PET-MR data of healthy volunteers (*n* = 67) to identify multiplex core nodes in the human brain. First, monoplex networks of structural, functional and metabolic connectivity were constructed, and consequently combined into a multiplex SC-FC-MC network by linking the same nodes categorically across layers. Taking into account the multiplex nature using a tensorial approach, we identified a set of core nodes in this multiplex network based on a combination of eigentensor centrality and overlapping degree. We introduced a coreness coefficient, which mitigates the effect of modeling parameters to obtain robust results. The proposed methodology was applied onto young and elderly healthy volunteers, where differences observed in the monoplex networks persisted in the multiplex as well. The multiplex core showed a decreased contribution to the default mode and salience network, while an increased contribution to the dorsal attention and somatosensory network was observed in the elderly population. Moreover, a clear distinction in eigentensor centrality was found between young and elderly healthy volunteers.

## Introduction

Over the past decade, graph theoretical approaches and computational network theory have proven their potential in neuroscience by modeling the brain as a complex network. Brain networks have become a rich area of research, which is also known as network neuroscience and ranges across different scales, from the microscale of interacting biomolecules up to the macroscale of social behavior (Sporns et al., 2004; Bullmore and Sporns, 2009; Craddock et al., 2018). In network neuroscience, the brain is modeled as a network (graph) usually consisting of elements representing brain regions (i.e., graph nodes) and their pairwise interconnections (edges). Based on different neuroimaging techniques, various ways of node interconnections are described. The two most common ways to model the brain as a graph are given by structural and functional brain connectivity (SC and FC, respectively). Structural networks are usually measured by diffusion weighted (DW) magnetic resonance imaging (MRI), which measures the diffusion rate of water molecules as a result of their interactions with tissues in the brain. By applying fiber tractography algorithms, the white matter pathways in the brain are reconstructed, which results in structural interconnections between the network nodes. Various fiber tractography algorithms have been developed to track the white matter pathways in the brain using DW-MRI, where recently the crossing fiber limitations of the diffusion tensor model were overcome by estimating the fiber density orientation function (fODF) using constrained spherical deconvolution (CSD) (Tournier et al., 2007). CSD relies on the principle that the DW signal is given by the spherical convolution of the fODF with the response function which represents the DW signal profile for a typical fiber population. Functional connectivity is defined as the temporal dependency of neuronal activity between anatomically separated brain regions and is typically estimated by functional time series measured with resting-state functional MRI (rs-fMRI) (Raichle and Raichle, 2001) which focuses on BOLD signal alterations during the resting state of the brain. Although most connectivity studies are focusing on structural and functional connectivity, methodological advances have moved network neuroscience toward the field of molecular connectivity, measured with positron emission tomography (PET), and in particular metabolic connectivity (MC) where brain metabolism is measured with ^18^F-FDG PET. Basically, molecular connectivity relies on the assessment of regional co-variation in PET tracer uptake across subjects, which is different from fMRI studies where the regional co-variation across time series of the BOLD signal is measured within the same subject. However, the main advantage of molecular connectivity is the availability of various neuroimaging tracers, which provide a very specific signal and thus allow to identify different, complementary molecular networks. Besides metabolic connectivity using ^18^F-FDG PET, connectivity studies targeting brain neurotransmission systems have emerged (Caminiti et al., 2017), and amyloid networks and patterns of tau have been identified to assess the connectivity-based pathological spreading across the brain during the time course of dementia of the Alzheimer's type (Pereira et al., 2018; Franzmeier et al., 2019).

Network modeling approaches have successfully unraveled interesting features in the brain, such as small-world topology, indicating an organization of the brain in highly clustered sub-networks combined with a high level of global connectivity (Brettschneider et al., 2013), and core-periphery organization, with the core being a connected group of nodes showing high centrality or importance in the network and thought to be fundamental to support integration of information (Feneberg et al., 2018). Many different metrics are available to measure the centrality of nodes within the network. However, most of the centrality measures are generally positive and rather highly correlated, with high scores for the core nodes for nearly all centrality measures (van der Burgh et al., 2019). With the increasing success of mapping brain networks and availability of multiple MR- and PET-based connectivity measures, the need for novel methodologies to unravel the structure and function of the brain at multiple spatial and temporal scales is emerging. The identification of a multiplex core-periphery organization has recently been proposed by Battiston et al., where structural and functional networks are merged into a multiplex (Cistaro et al., 2012). These researchers hypothesized that integrating information from both structural and functional networks give a more accurate estimate of the regions that contribute to the core of the human cortex. Although they combine features from different layers into one metric describing core-periphery organization, the underlying, high complexity of the multiplex approach was not fully elaborated. More recently, a multilayer network approach has been developed which provides a mathematical framework to model and analyze complex data using multivariate and multi-scale information (van den Heuvel et al., 2008; van den Heuvel and Sporns, 2011). More specifically, this approach uses a tensorial framework instead of adjacency matrices (Oldham et al., 2019) which are useful to describe traditional single layer networks, but are unable to capture to complex architecture of multilayer networks.

In this work, we use hybrid PET-MR data of healthy controls to construct a representation of brain networks at three different levels of connectivity. Structural, functional, and metabolic networks are constructed based on DW-MRI, rs-fMRI and ^18^F-FDG PET, respectively. First, we introduce a novel approach to define a multiplex network and identify the multiplex core, taking into account the complex architecture of multilayer networks by using a tensorial framework. This multiplex network approach allows to identify novel network metrics, which may provide additional information that might be undetected by monoplex metrics. Second, we apply this method to the SC—FC—MC multiplex and investigate the effect of aging on the multiplex core architecture in the brain. On the one hand, we study whether differences in the monoplex networks persist in the multiplex network as well, while, on the other hand, we investigate if a multiplex metric enables to find a difference between young and elderly healthy volunteers.

## Methods

### PET-MR data

Sixty-seven healthy volunteers (mean age: 51.1 ± 16.4 years, range 20–82 years, almost uniformly distributed) were recruited prospectively between December 2015 and February 2017. The main exclusion criteria consisted of major internal pathology, diabetes mellitus, cancer, absence of a first-degree relative with dementia, history of important neurological and/or psychiatric disorders or substance abuse or pre-study use of centrally acting medication. All subjects underwent a complete neurological examination, performed by a board-certified physician, had a mini-mental state examination (MMSE) score ≥ 28 and their index on the becks depression inventory (BDI) was ≤ 9. The study was approved by the ethics committee of the University Hospital Leuven (study number s58571—Belgian Registration Number b322201526273) and was conducted in full accordance with the latest version of the declaration of Helsinki. All participants provided written informed consent before inclusion in the study.

^18^F-FDG was administered by intravenous injection of 151.9 ± 9.8 MBq. All subjects underwent simultaneous FDG PET and MR scanning on a hybrid 3T Signa PET-MR scanner (GE healthcare, Chicago, IL, USA). List mode images were acquired upon tracer injection in the scanner for 60 min, from which static (40–60 min pi) data were derived. The first 15 min of the simultaneous scan, no MR sequences were applied in order not to invoke primary auditory cortex activation. MR image acquisition was performed using an 8-channel phased-array coil. In addition to a whole brain volumetric T1-weighted image (3D BRAVO, TR/TE = 8.5/3.2 ms, 1 × 1 × 1 mm voxel size) and a fluid-attenuated inversion recovery (FLAIR) image (3D CUBE, TR/TE = 8,500/130 ms, 1 × 1 × 1.4 mm voxel size) were collected. Resting-state data in eyes-open condition were acquired with TR/TE = 1.7 s/2 ms, flip angle = 90, voxel size = 3.6 × 3.6 × 4 mm. DTI and reverse phased DTI were acquired using a b-value of 1,500 s/mm^2^, applied along 48 uniformly distributed directions (TR/TE = 12,000/85 ms, 2.5 mm isotropic voxel size mm). Default vendor-based MRAC (MR-based attenuation correction, v1.0) corrected PET images were reconstructed using ordered subset expectation maximization (OSEM) with six iterations and 28 subsets, and post-smoothed with a 3 mm isotropic Gaussian filter.

Patients showing too much movement were excluded from the start, since this can have a large impact on rs-fMRI data and subsequent analysis, with exclusion criterium: mean framewise displacement higher than 0.3 mm.

To investigate the effect of aging, we selected a “young” (*n* = 26) and “elderly” (*n* = 28) population by setting an age threshold at age ≤ 45 and age ≥ 55, respectively and identified the core nodes for both groups.

### Brain connectivity

Both structural, functional, and metabolic networks consisted of 100 cortical nodes, defined by regions of interest (ROIs) obtained with a Schaefer parcellation scheme (Kivelä et al., 2014). As such, each network was represented by a 100 × 100 adjacency matrix, describing the connectivity or edge weights between each pair of nodes. SC and FC connectomes for a specific population were calculated as the average weighted network across the population. For each modality, the different type of information and different way of network construction resulted in an unequal level of network sparsity of the corresponding weighted adjacency matrix. Therefore, all networks were binarized to ensure that the connectivity density, i.e., the total number of edges, of each network was equal. However, since the choice of binarizing threshold is rather arbitrary, results were averaged across a full range of connectivity densities (from 10% up to 50%, stepsize: 1%).

#### Structural connectivity

Diffusion images were processed using MRtrix3 (Boccaletti et al., 2014) and the FMRIB Software Library (De Domenico et al., 2014). Preprocessing of the diffusion MRI data included denoising, Gibbs ringing removal, correction for EPI susceptibility, eddy-current-induced distortions, gradient-nonlinearities, and subject motion. From the corrected diffusion data, response functions for single-fiber white matter (WM), gray matter (GM) and cerebrospinal fluid (CSF) were estimated using an unsupervised method (Schaefer et al., 2018). Single-shell 3-tissue constrained spherical deconvolution (SS3T-CSD) was performed to obtain the fiber orientation distributions (FODs) for WM, GM and CSF (Tournier et al., 2012) using MRtrix3Tissue (https://3Tissue.github.io), a fork of MRtrix3 (Boccaletti et al., 2014). Consequently, the FODs were used to conduct deterministic tractography using the Fiber Assignment by Continuous Tracking (FACT) algorithm, which tracks the trajectory of white matter tracts by propagating streamlines along the primary direction of water diffusion at each voxel (Jenkinson et al., 2012). Anatomically Constrained Tractography was performed alongside FACT using the tissue-segmented T1-weighted image to ensure that the generated streamlines were biologically accurate (D'Hollander Tijs et al., 2019). Whole brain tractograms were re-weighted using Spherically Informed Filtering of Tractograms 2 (SIFT2) (D'Hollander Tijs, 2016), which adjusted the streamline weights to represent the underlying diffusion signal more accurately. For each subject, the tractogram and Schaefer parcellation were combined to produce a subject-specific structural connectome. The corresponding edge weights were defined by the number of streamlines between two nodes, normalized by the volumes of both regions represented by the two nodes such that each value of the SC matrix reflected the density of the white matter streamlines between the corresponding two nodes.

#### Functional connectivity

Preprocessing of the BOLD time-series was performed using fmriprep version 1.5.10 (Esteban et al., 2019). Each T1-weighted (T1w) volume was corrected for intensity non-uniformity using N4BiasFieldCorrection [217] and skull-stripped using Advanced Normalization Tools (ANTs) (De Leener et al., 2017). Spatial normalization to the ICBM 152 Non-linear Asymmetrical template (version 2009c) (Smith et al., 2012) was performed through non-linear registration with the ANTs registration tool using brain-extracted versions of both T1w volume and template. Brain-tissue segmentation of CSF, WM and GM was performed on the brain-extracted T1w volume using FSL (Smith et al., 2015). Functional data were slice-time corrected using AFNI (3dTshift) (Tustison et al., 2010), and realigned to a mean reference image using FSL (mcflirt) (Fonov et al., 2009). Fieldmap-less distortion correction was performed by co-registering the functional image to the intensity-inverted T1w image (Zhang et al., 2001), constrained with an EPI distortion atlas (Cox and Hyde, 1997) and implemented with ANTs (antsRegistration). This was followed by co-registration to the corresponding T1w volume using boundary-based image registration (Jenkinson et al., 2002) with nine degrees of freedom. Framewise displacement was calculated for each functional run (Wang et al., 2017). The non-aggressive variant of ICA-based Automatic Removal of Motion Artifacts (AROMA) was used to generate and remove noise components from the fmriprep-processed output (Treiber et al., 2016). Subsequently, mean WM, mean CSF and global mean signals were calculated and regressed out in a single step using least squared regression. Finally, the data were filtered with a high-frequency bandpass filter of (0.01, 0.1 Hz) to exclude confounding high-frequency content. After pre-processing the BOLD rs-fMRI time-series for each individual, edge weights for the functional networks were calculated by the Pearson correlation coefficient between the average time-series in each ROI.

#### Metabolic connectivity

For each subject, the ^18^F-FDG uptake was normalized by proportional scaling, i.e., dividing the uptake by the total uptake in the gray matter, after smoothing the data with a Gaussian kernel with a Full Width Half Maximum (FWHM) of 8 mm. Although different strategies exist to define metabolic connectivity networks, all these approaches rely on the assessment of regional co-variation in ^18^F-FDG uptake across subjects, which is different from the subject-specific approaches to define structural and functional connectivity. We applied Sparse Inverse Covariance Estimation (SICE) (Sala et al., 2017), also known as Gaussian graphical modeling. Basically, SICE imposes a sparsity constraint on the maximum likelihood solution of the inverse covariance (IC) matrix under the assumption of a Gaussian model, which means that the sample size can be less than the number of brain regions modeled. Since the brain network organization is assumed to be sparse (Rubinov and Sporns, 2010), SICE is considered as a valid approach to model metabolic brain connectivity. Although SICE has proven to be an effective tool for identifying the structure of an IC matrix, its use is not recommended to estimate the magnitude of the non-zero entries in case of weighted adjacency matrices (Sala et al., 2017). However, since we work with binary instead of weighted matrices, SICE is an appropriate choice for our approach to estimate the zero and non-zero entries of the IC matrix.

### Multilayer network

A multilayer network consists of several classical networks, each layer encoding a specific type of information. Since multilayer networks can no longer be represented by classical adjacency matrices, we used a tensor formalism to describe these networks. More specifically, we defined a multilayer adjacency tensor of *N* nodes and *L* layers with components given by $M_{i\alpha }^{j\beta }$, encoding the connectivity between node *i* in layer α and node *j* in layer β (*i, j* = 1, 2, …, *N*; α, β = 1, 2, …, *L*). To easily represent the tensor, we used the standard approach of flattening this rank-4 tensor into a rank-2 tensor, also known as the supra-adjacency matrix, with the diagonal blocks encoding the inter-layer connectivity. For our three-modal PET-MR data, the multilayer network consisted of three layers, i.e., a structural, functional and metabolic connectivity layer, resulting in a SC—FC—MC multilayer network. In order to naturally extend classical network metrics, the different layers should be interconnected since otherwise the analysis of the multilayer adjacency tensor comes down to analyzing each layer separately. To combine individual layers, links are added between corresponding nodes across layers, either ordinally by linking corresponding nodes between adjacent layers only, or categorically, by linking a node to the corresponding nodes across all layers. We implemented the latter option since ordinally linking assumes that neighboring networks are prioritized.

Since core nodes tend to score high on nearly all centrality measures, we focused on a combination of degree centrality (DC) and eigenvector centrality (EC) for defining the core nodes in the multilayer networks. In a single layer network with adjacency matrix *A*, the DC of node *i* is given by its number of connections, where the EC of node *i* represented by *x*_*i*_ is defined as:


(1) $$\begin{array}{l} x_{i}=\frac{1}{\lambda }\underset{k}{\sum }A_{ki}x_{k} \end{array}$$

As a result, EC measures a node's importance while taking into account the importance of its neighbors. This can be written in matrix form *Ax* = λ*x*, which is the eigenvector equation of *A*. Since the adjacency matrix *A* is positive definite, the Perron-Frobenius theorem states that there exist a unique and positive eigenvector corresponding to the leading eigenvalue λ_1_, yielding the EC values for all nodes. EC values were calculated by the power iteration method, which makes sense intuitively. All nodes start with equal EC, but as the computation progresses, nodes with more edges start gaining importance, which mainly propagates to the nodes to which they are connected. After several iterations, EC values stabilize (error tolerance of 1e-5), resulting in the final EC estimates.

Both DC and EC measures for nodes in monoplex network were generalized toward multilayer networks. The (scalar) degree of a node in a multilayer network is either given by the degree of the aggregated topological network, i.e., the union of the monoplexes, or by the overlapping (summed) degree $o_{i}=\underset{\alpha }{\sum }k_{i}^{\alpha }$, where $k_{i}^{\alpha }$ is the degree of node *i* in layer α. Since both degree measures tend to be highly correlated, the overlapping degree centrality (ODC) was used. On the other hand, EC generalization is less trivial and there are several ways to do so (Greve and Fischl, 2009). The most elegant way is to rewrite the eigenvalue equation using an equivalent tensor formulation (single layer):


(2) $$\begin{array}{l} A_{i}^{j}x_{j}=\lambda_{1}x_{i} \end{array}$$

using the Einstein summation convention $A_{i}^{j}x_{j}\equiv \underset{j}{\sum }A_{i}^{j}x_{j}$. As a result, the adjacency matrix is now given by the rank-2 tensor $A_{i}^{j}$ containing one contravariant and one covariant component. The generalization of the above equation to the multilayer case with adjacency tensor $M_{i\alpha }^{j\beta }$ is then given by:


(3) $$\begin{array}{l} M_{i\alpha }^{j\beta }\Theta_{j\beta }=\lambda_{1}\Theta_{i\alpha } \end{array}$$

with λ_1_ the leading eigenvalue and Θ_*iα*_ the corresponding eigentensor. This equation can easily be solved using the supra-adjacency matrix formulation:


(4) $$\begin{array}{l} \left(\begin{array}{ccc} M^{1} & wI_{N} & wI_{N}\\ wI_{N} & M^{2} & wI_{N}\\ wI_{N} & wI_{N} & M^{3} \end{array}\right)\left(\begin{array}{c} \Theta^{1\;}\\ \Theta^{2\;}\\ \Theta^{3\;} \end{array}\right)=\lambda_{1}\left(\begin{array}{c} \Theta^{1\;}\\ \Theta^{2\;}\\ \Theta^{3\;} \end{array}\right) \end{array}$$

with *I*_*N*_ the unity matrix of size *N* × *N*, *M*^α^ corresponds to the single-layer adjacency matrix of layer α, $\Theta_{i}^{\alpha \;}\equiv \Theta_{i\alpha }$ encodes the eigentensor centrality (ETC) of each node *i* in layer α while accounting for the whole interconnected structure, and *w* is an intra-layer weight factor. The ETC value θ_*i*_ of each node is found by contracting Θ_*i*_ with the rank-1 tensors *u*^α^ with all components equal to 1, i.e., $\theta_{i}=\Theta_{i\alpha }u^{\alpha }$. The choice of this aggregation corresponds to a maximum entropy principle, which is a valid choice when all layers are considered equally important (Power et al., 2014). The weight factor *w* is chosen such that the total number of inter-layer connections is equal to the intra-layer connections, i.e.,


(5) $$\begin{array}{l} w=\frac{3\gamma C_{N}^{2}}{6N}=\frac{\gamma \left(N-1\right)}{4} \end{array}$$

where *N* is the number of nodes, $C_{N}^{2}$ is the number of 2-combinations out of N, i.e., $C_{N}^{2}=N\left(N-1\right)/2$, and γ is the connectivity density of the monoplex networks.

### Core selection

At each binarizing threshold between 0.1 and 0.5 (step 0.01), we identified a set of core nodes by selecting these nodes scoring high, i.e., a fraction δ of the standard deviation σ above the mean value μ (μ+δ*σ), on both DC/ODC and EC/ETC (depending on the monoplex/multiplex nature of the network). Since the definition of “scoring high” depends on the parameter δ, and therefore is rather arbitrary, we calculated a set of core nodes for different δ values (from 0.4 to 1.6, stepsize 0.2). In this way, the coreness coefficient *C*_*i*_ of each node *i* is calculated as the normalized frequency (across binarizing thresholds and δ values) of being part of the core. To make the interpretation more convenient, we colored the top 15% of the most central nodes in a different color.

To quantify the similarity between the cores of layer α and layer β, the core similarity coefficient is defined as:


(6) $$\begin{array}{l} S_{c}=\frac{I_{c}^{\alpha \beta }}{N_{c}^{\alpha }}\;\left(0<\;S_{c}<1\right) \end{array}$$

where $I_{c}^{\alpha \beta }$ is the number of nodes which are part of the core of both layer α and β, and $N_{c}^{\alpha }$ is the total number of nodes in layer α (De Domenico, 2017).

First, the structural (SC), functional (FC) and metabolic (MC) connectomes are calculated for the average population. Consequently, the corresponding monoplex cores are identified based on the combined (DC, EC) measure, and the multiplex core is obtained by selecting nodes scoring high on both ODC and ETC. SC and FC population connectomes were obtained by calculating the average weighted SC and FC networks, respectively.

## Results

### Average brain

**Figure 1:**
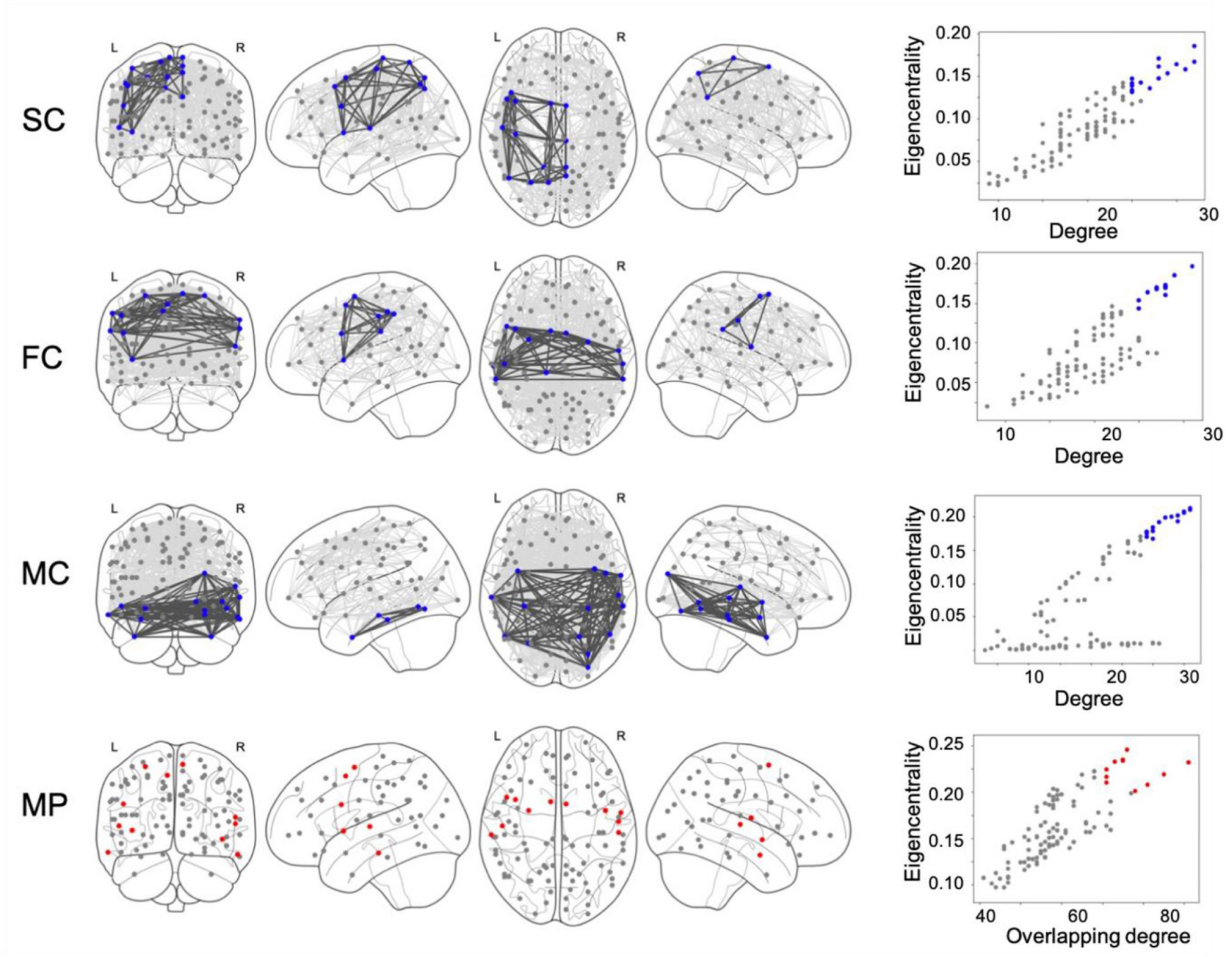
Structural connectivity (SC), functional connectivity (FC) and metabolic connectivity (MC) networks are calculated at 20% network density and shown together with the corresponding core nodes (marked in blue). Core nodes are identified as nodes scoring high, i.e., one standard deviation (std) above the mean, on both degree and eigenvector centrality. Multiplex (MP) core nodes, marked in red, are analogously defined by one std above the mean of overlapping degree and eigentensor centrality.

As an example, the network connectivity and core nodes at 20% network density are shown in Figure 1, where core nodes are selected as the nodes having one standard deviation above the mean value on both DC/ODC and EC/ETC (α = 1).

**Figure 2:**
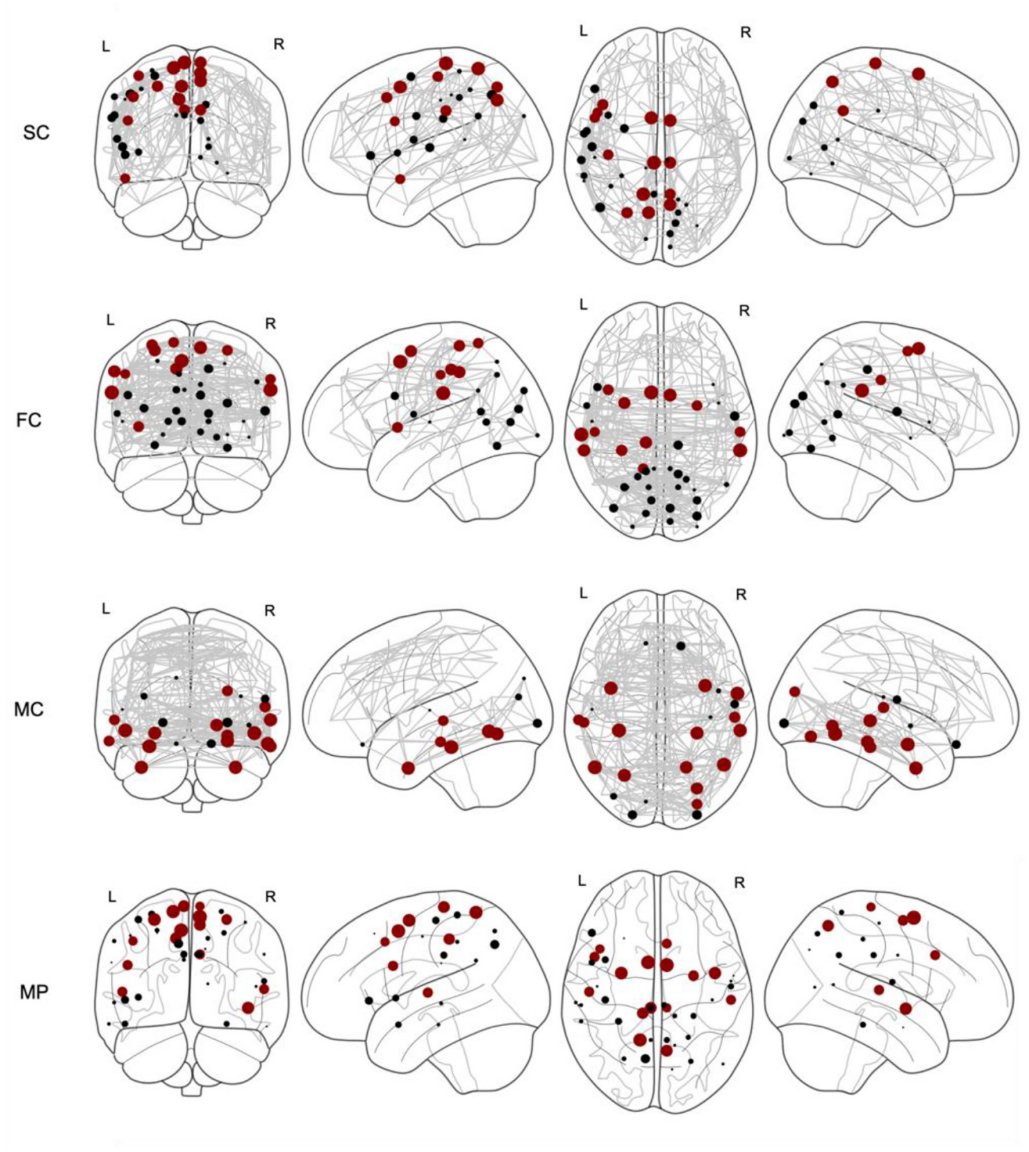
Core nodes (marked as dots) for structural connectivity (SC), functional connectivity (FC), metabolic connectivity (MC) and multiplex (MP) networks. The size of each marked node corresponds to the coreness coefficient. For each network, the top 15% nodes with highest coreness coefficient are marked in red.

**Figure 3:**
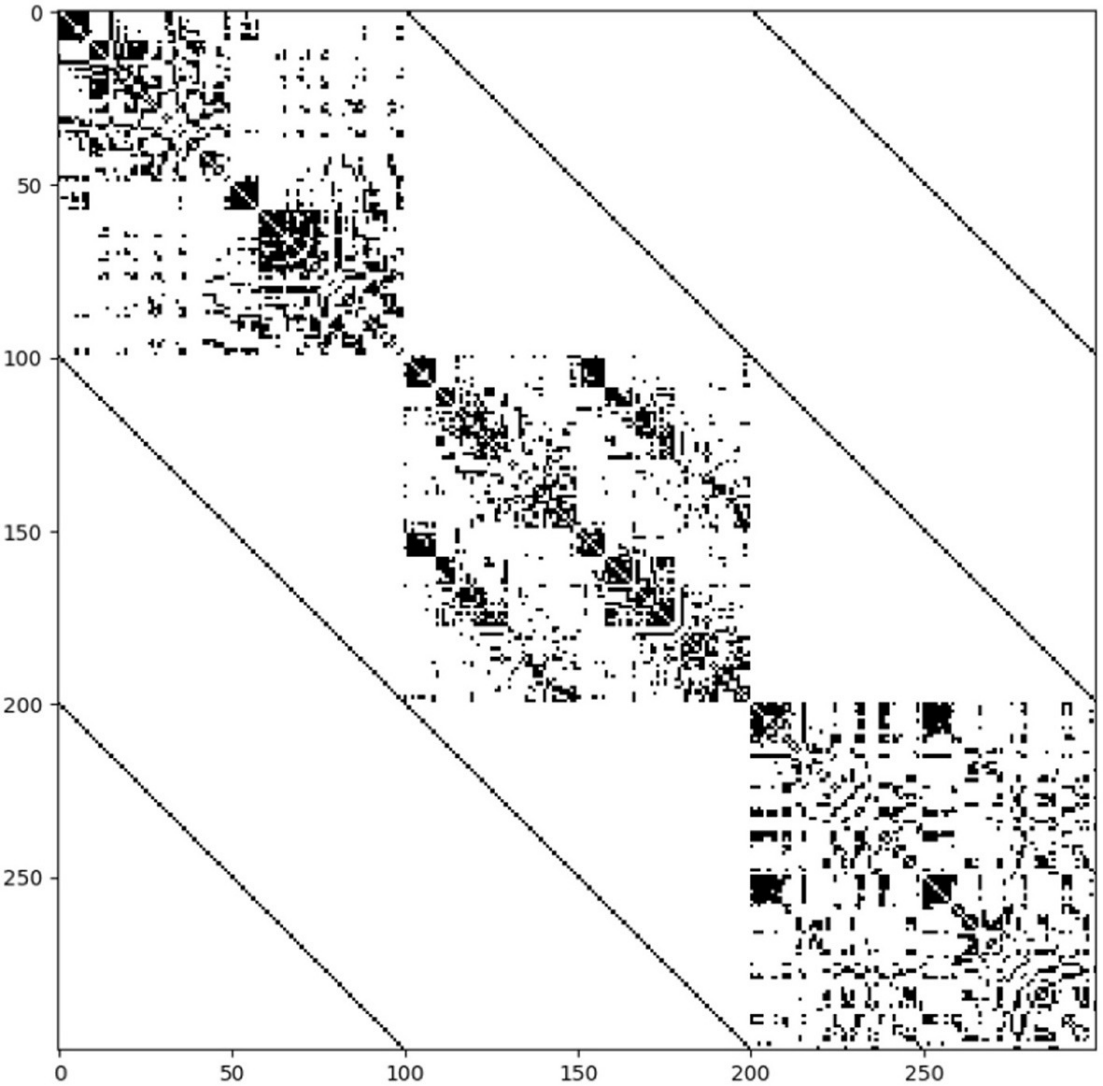
Structure of the supra-adjacency matrix of the multilayer SC—FC—MC network (connections are indicated in black) at 20% connectivity density. The matrix consists of three main diagonal blocks, corresponding to the structural **(upper left)**, functional **(middle)** and metabolic **(lower right)** connectivity layers, respectively. The off-diagonal lines represent the connections between nodes across layers, which are linked categorically, and are weighted by a factor five (approx.) to balance the block diagonal structure.

The coreness coefficient *C*_*i*_, taking into account a full range of binarizing thresholds, is shown in Figure 2, where the size of the nodes corresponds to *C*_*i*_. For each connectome, the top 15% of nodes scoring highest on the coreness coefficient *C*_*i*_ are marked in red. Thereafter, a multilayer SC—FC—MC network is constructed, as illustrated by the supra-adjacency matrix given in Figure 3 (20% network density). The matrix consists of three main diagonal blocks, corresponding to the structural (upper left), functional (middle) and metabolic (lower right) connectivity layers, respectively. Nodes across layers are linked categorically, which is illustrated by the non-diagonal lines in Figure 3. The multilayer core nodes are identified by a combination of high values for both overlapping degree and eigentensor centrality.

For each single-layer network, we selected the top 15% nodes with highest coreness coefficient (as marked in red in Figure 2) and calculated the core similarity for this selection of nodes with respect to the multiplex, which resulted in 40% for SC, 40% for FC and 13% for MC. This “selection” of core nodes (top 15%) with the corresponding coreness coefficient is given in the Supplementary material. The percentage of the nodes of this selected core which are part of each network, as defined by the Schaefer atlas, is given in Table 1. These data demonstrated that, for the older participants, less regions of the salient network were being ranked amongst the regions with highest coreness coefficient compared to the younger participants, while for the dorsal attention network, more regions are ranked amongst the regions with highest coreness coefficient for the older group compared to the younger group (see also Supplementary Table S1).

**Table 1:** Percentage of the nodes within the core (selection of top 15%) which are part of each network, as defined by the Schaefer atlas, for the average (A), young (Y) and elderly (O) population.

	**SC (%)**			**FC (%)**			**MC (%)**			**MP (%)**		
	**A**	**Y**	**O**	**A**	**Y**	**O**	**A**	**Y**	**O**	**A**	**Y**	**O**
Visual network	0	0	0	0	0	0	33	33	33	0	0	0
Somatomotor network	27	27	27	13	13	13	13	7	27	27	20	40
Dorsal attention network	27	27	27	33	27	40	7	7	7	27	27	33
Salience network	13	13	13	47	53	40	7	7	7	33	33	20
Limbic network	0	0	0	0	0	0	13	13	13	0	0	0
Control network	27	27	27	7	7	7	0	0	0	7	7	7
Default mode network	7	7	7	0	0	0	27	33	13	7	14	0

### Aging

**Figure 4:**
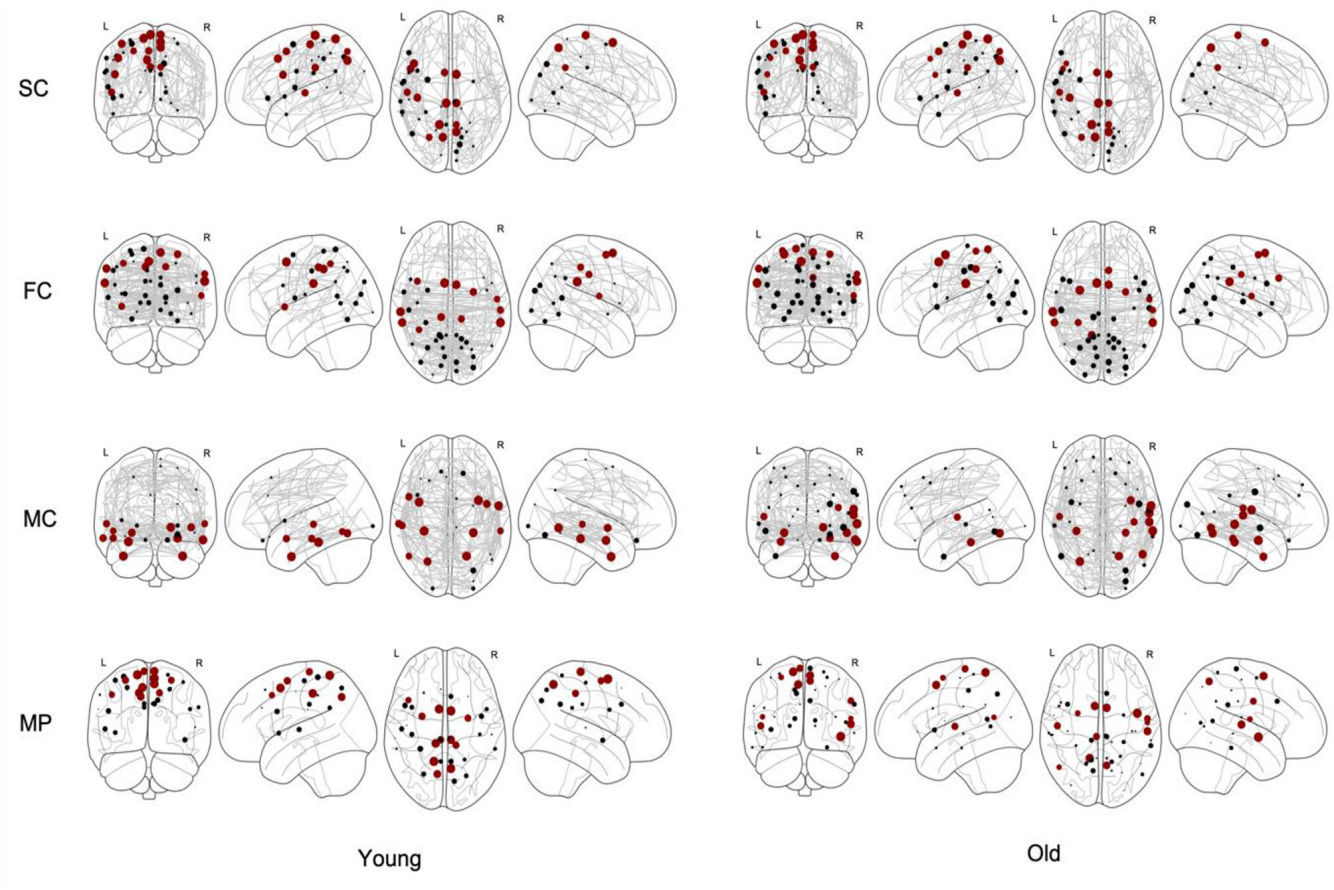
Core nodes (marked as dots) for young and old population for structural connectivity (SC), functional connectivity (FC), metabolic connectivity (MC) and multiplex (MP) networks. The size of each marked node corresponds to its normalized coreness coefficient. The top 15% of the nodes with highest coreness coefficient are marked in red.

We identified the core nodes for both the young and elderly population, as illustrated in Figure 4, where the top 15% of most central nodes, i.e., with highest coreness coefficient, is marked in red. This selection of core nodes with the corresponding coreness coefficient is given in the Supplementary material. The percentage of the nodes of this selected core which are part of each of the seven functional networks (as defined by the atlas) for both young and old population is given in Table 1.

**Figure 5:**
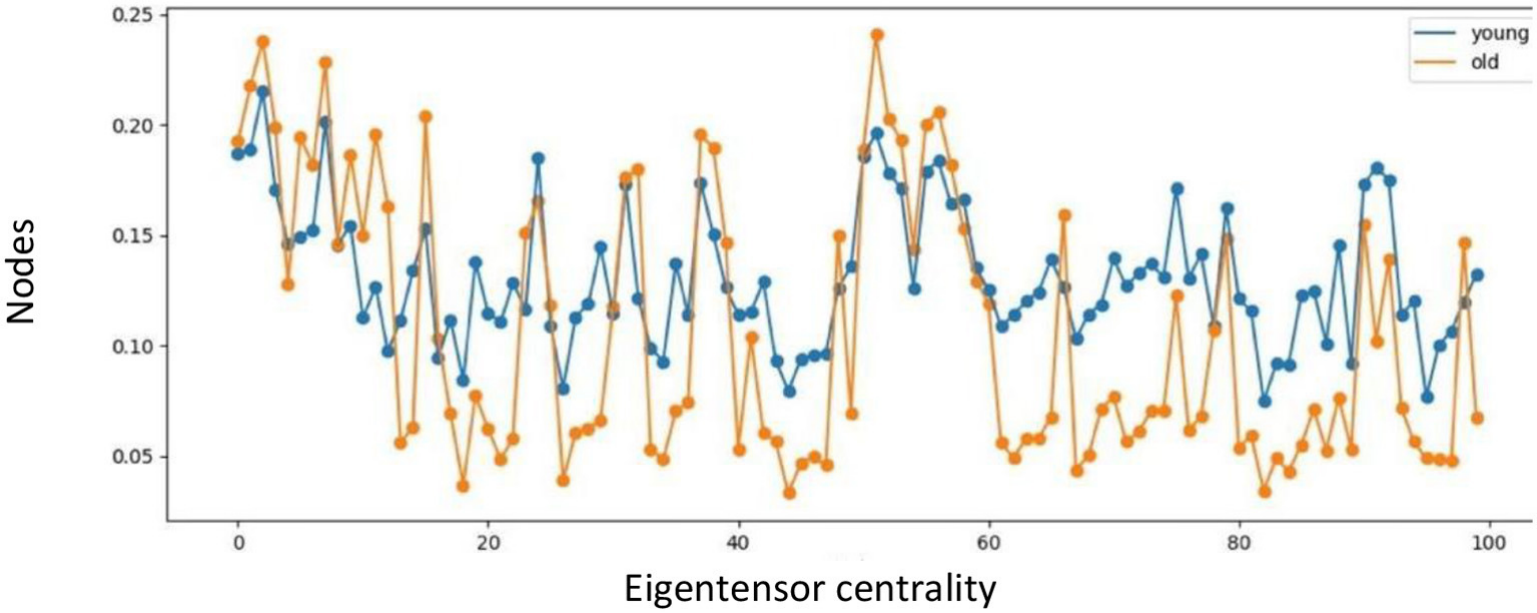
Eigentensor centrality for young and old population.

The eigentensor centrality of the multiplex network is averaged across the range of binarizing connectivity thresholds, and higher for most nodes in the young population compared to the old population, as illustrated in Figure 5. In 70% of the nodes, the eigentensor centrality of the young population is higher compared to the old population, whereof in 60% the relative difference is 30% or higher. In contrast, of the 30% nodes where the eigentensor centrality of the old population is higher, only 3% shows a relative difference of at least 30%. The core similarity coefficient between young and old age group resulted in 0.93, 0.8, 0.69, and 0.5 for the SC, FC, MC and multiplex network, respectively.

## Discussion

### Technical aspects

In this work, we introduced a novel approach to identify a multilayer core in the human brain by taking into account the complex architecture of a multilayer network using a tensorial framework. We used PET/MR data of healthy controls to construct brain networks at three different levels of connectivity. More specifically, structural connectivity (SC), functional connectivity (FC) and metabolic connectivity (MC) networks were constructed based on diffusion weighted MRI, rs-fMRI and ^18^F-FDG PET, respectively. The multilayer core in the resulting SC—FC—MC multiplex was identified by selecting the nodes scoring high on both overlapping degree and eigentensor centrality. We focused on the combination of these two centrality measures since core nodes in single-layer networks tend to score high on nearly all centrality measures (van der Burgh et al., 2019). Node degree is commonly used to identify brain hubs, while eigenvector centrality has an elegant generalization to eigentensor centrality of multilayer networks using a tensorial framework. Usually, nodes with a high eigenvector centrality are important in the sense that they are linked to other nodes with high eigenvector centrality and therefore represent highly clustered nodes. Moreover, our findings demonstrated that both the overlapping degree and eigentensor centrality rank distribution showed an exponential behavior at both ends of the distribution (see Supplementary material). Therefore, only a small fraction of nodes had a significantly large value for both centrality measures. Hence, the combination of overlapping degree and eigentensor centrality in a multilayer network ascertained the most central nodes which should be considered the core nodes within the network.

Understanding the interplay between brain structure, function and molecular organization is an ongoing challenge in neuroscience. For example, SC—FC multiplex networks were recently derived to identify multiplex motifs (Battiston et al., 2018), which represent specific subgraphs of reduced size that play a fundamental role in the stability of the underlying system and several corresponding functions (Solá et al., 2013). However, at the mesoscale level, the detection of core-periphery organization in multiplex brain networks has been poorly explored. Recently, Battiston et al. proposed a framework to detect core nodes in multiplex networks (De Domenico, 2017) based on a scalar richness coefficient which was defined by a weighted sum of single-layer degree information but didn't really allow to take into account the multiplex nature of the SC—FC network. In contrast to their method, our approach took into account the complex architecture of a multiplex network for defining the core structure by linking different layers together categorically, as illustrated in Figure 3, and using a tensorial framework to define eigentensor centrality. Moreover, to the best of our knowledge, our study combined for the first time structural, functional, and metabolic information, derived from PET/MR imaging, to identify a multiplex core organization in the human brain.

During network construction, the weight matrices for the SC, FC, and MC networks were binarized since the sparsity level of the three different networks is not equal. After a different binary threshold was applied for each type of network to obtain the same network density level for all network types, a proper integration of the three different networks into a multiplex was made. Since the choice of network density was rather arbitrary, a full range of densities was considered, yielding a core structure for each network density level. However, the core at each network density level was defined by selecting the nodes having both a high overlapping degree and eigentensor centrality. As this definition of “high” was also arbitrary, we considered a range of overlapping degree and eigentensor centrality values. As such, the coreness coefficient for each node was introduced, being the normalized frequency (across network densities and core selection range) of each node being part of the core. Therefore, a robust metric is obtained which is less independent of modeling parameters.

### Clinical relevance

Based on the results shown in Table 1 and Figure 4, we observed no differences in the core structure of the SC networks between the young and elderly population. However, we found a shift in core organization in the FC and MC networks between young and old. More specifically, in the FC networks, we observed an increase of core nodes with aging in the dorsal attention network together with a decrease in the salience network, whereas in the MC networks, an increase of core nodes in the somatomotor cortex was found with aging together with decrease in the default mode network. The reason behind the observed increase of core nodes in the somatomotor cortex might be explained by a compensatory mechanism due to a decreased integrative capacity (Milo et al., 2002). These findings are in line with literature, with changes in the default mode network and somatomotor cortex being detected by the core organization of the MC networks which confirms the hypothesis that functional changes with aging precede structural ones (Solá et al., 2013; De Domenico et al., 2015). In addition, these results demonstrate the complementarity of FC and MC metrics, although literature data have also shown that functional connections determined by rs-fMRI are related to glucose metabolism (Palombit et al., 2022). On the other hand, the correlation strength between the spatial distributions of PET and rs-fMRI-derived metrics has proven to be spatially heterogeneous across both anatomic regions and functional networks, with lowest correlation strength in the limbic network, and strongest correlation for the default-mode network (Aiello et al., 2015). This coupling between glucose metabolism and functional connectivity, which was observed in healthy aging, was substantially reduced in patients with amnestic mild cognitive impairment and Alzheimer's disease, suggesting that changes in glucose utilization could be linked to a reduced communication among brain regions impacted by the underlying pathological process (Marchitelli et al., 2018). However, findings of these studies are based on individual ^18^F-FDG PET measurements of regional glucose metabolism without considering MC, while this study considers a population-based MC network, such that comparing results is not straightforward. For future research purposes, novel approaches such as the Kullback-Leibler divergence similarity estimation (KLSE) can be considered to generate an individual brain metabolic network for a single subject using static ^18^F-FDG PET imaging. This technique assumes that brain regions with similar glucose metabolism are highly interconnected while brain regions with differences in glucose metabolism have a lower connectivity strength. Using these metabolic connectivity strengths, the approach successfully predicted individual risk of progression from Mild Cognitive Impairment (MCI) to Alzheimer's Disease (AD) (Wang et al., 2020) while age age-related effects on graph-based connectivity measures using KLSE have also been evaluated (Mertens et al., 2022). However, the KLSE approach compares the intra-regional ^18^F-FDG distribution between different brain regions within a single subject such that it provides a quantitative representation of the ^18^F-FDG distribution throughout the brain and different subnetworks with a high average metabolic strength between nodes corresponding to a rather homogeneous ^18^F-FDG uptake in the corresponding brain regions. As such, the KLSE approach should be considered as a different but potentially complementary approach to MC estimates across subjects using correlation measures, as was used in this and other studies (Arnemann et al., 2018; Huang et al., 2022), with the latter approach being more in line with standard techniques to estimate SC and FC.

The differences in the core organization observed between young and old in the FC and MC networks were confirmed by the multiplex network (Table 1). Moreover, we found a clear difference in eigentensor centrality derived from the multiplex network between both age populations, as illustrated in Figure 5. Therefore, multiplex networks and corresponding metrics might be considered as advanced biomarker(s) in aging and neurodegenerative disorders, as they integrate effects detected by SC, FC, and MC and therefore could improve diagnosis and patient stratification. However, more research is needed to test the discriminative potential of multiplex networks and corresponding advanced metrics in aging and neurodegenerative disorders.

### Limitations

The main limitation of this study is the population-based MC measure which relies on the assessment of regional co-variation in ^18^F-FDG uptake across subjects, while SC and FC which are calculated on the individual subject level and then averaged. Because of this group-based correlation approach for MC, two groups with a different age range needed to be considered to evaluate the age dependency of MC metrics, which generated only one estimate for each connectivity metric per group, therefore limiting the evaluation of age dependent effects to an observational description of changes.

## Conclusion

Based on PET/MR imaging, monoplex networks of structural, functional, and metabolic connectivity were first constructed, and consequently combined into a multiplex SC-FC-MC network by linking the same nodes categorically across layers. Based on a combination of eigentensor centrality and overlapping degree, we identified the core nodes in this multiplex network, while taking into account the multiplex nature using a tensorial representation. The proposed methodology was applied to young and elderly healthy volunteers, where differences observed in the monoplex networks were confirmed by the multiplex approach. Furthermore, a clear distinction in eigentensor centrality was found between young and old healthy volunteers. These findings demonstrate the potential of multiplex networks as an integrative approach to capture the relevant information in hybrid neuroimaging data.

## Data availability statement

The original contributions presented in the study are included in the article/Supplementary material, further inquiries can be directed to the corresponding author.

## Ethics statement

The studies involving human participants were reviewed and approved by Ethics Committee of the University Hospital Leuven. Study number S58571. Belgian registration number B322201526273. The study was in full accordance with the latest version of the Declaration of Helsinki. The patients/participants provided their written informed consent to participate in this study.

## Author contributions

MD performed the data analysis, model development, and writing of the manuscript. KV and MK have critically contributed to the manuscript and have approved the final content. All authors contributed to the article and approved the submitted version.
