# Supplementary material for: Multiplex core of the human brain using structural, functional and metabolic connectivity derived from hybrid PET-MR imaging
Source: Front Neuroimaging. 2023 Aug 14;2:1115965. doi: 10.3389/fnimg.2023.1115965 (PMC10461102; doi:10.3389/fnimg.2023.1115965)
Supplement: Supplementary file 1 [file Data_Sheet_1.docx]

**Supplementary material**

Table S1. Top 15% of the regions with highest coreness coefficient.

|  | Average | $C_{i}$(%) | Young | $C_{i}$(%) | Old | $C_{i}$(%) |
| --- | --- | --- | --- | --- | --- | --- |
| SC | LH_SomMot_8  LH_DorsAttn_Post_5  LH_Cont_pCun_1  RH_DorsAttn_Post_5  RH_SalVentAttn_Med_3  RH_Default_pCunPCC_2  RH_SomMot_6  LH_Cont_Cing_1  LH_SalVentAttn_Med_2  RH_Cont_pCun_1  LH_SomMot_6  LH_DorsAttn_Post_4  LH_Cont_Par_2  RH_Cont_Cing_1  LH_SomMot_1 | 100  97  92  88  79  75  74  74  73  55  51  51  50  56  45 | LH_SomMot_8  LH_DorsAttn_Post_5  LH_Cont_pCun_1  RH_DorsAttn_Post_5  RH_SalVentAttn_Med_3  RH_SomMot_6  RH_Default_pCunPCC_2  LH_Cont_Cing_1  LH_SalVentAttn_Med_2  RH_Cont_pCun_1  LH_SomMot_6  LH_Cont_PFCl_4  LH_SomMot_1  LH_DorsAttn_PrCv_1  LH_DorsAttn_Post_4 | 100  96  94  93  86  80  72  67  68  61  57  54  51  48  47 | LH_SomMot_8  LH_DorsAttn_Post_5  LH_Cont_pCun_1  RH_DorsAttn_Post_5  RH_Default_pCunPCC_2  RH_SalVentAttn_Med_3  RH_SomMot_6  LH_SalVentAttn_Med_2  LH_Cont_Cing_1  LH_SomMot_6  RH_Cont_pCun_1  LH_SomMot_1  LH_Cont_Par_2  LH_DorsAttn_Post_4  LH_DorsAttn_PrCv_1 | 100  96  94  88  75  65  65  65  62  59  53  48  46  46  41 |
| FC | RH_SalVentAttn_TempOccPar_2  LH_SalVentAttn_ParOper_1  RH_SalVentAttn_Med_2  LH_SalVentAttn_Med_3  RH_SalVentAttn_Med_1  RH_Cont_Par_1  RH_DorsAttn_Post_5  RH_DorsAttn_FEF_1  LH_SalVentAttn_Med_2  RH_SomMot_7  LH_DorsAttn_Post_2  RH_SalVentAttn_FrOperIns_1  LH_SomMot_3  LH_DorsAttn_FEF_1  RH_DorsAttn_Post_2 | 100  99  96  92  83  70  69  61  60  57  56  50  47  46  44 | LH_SalVentAttn_ParOper_1  LH_SalVentAttn_Med_3  RH_SalVentAttn_Med_2  RH_SalVentAttn_Med_1  RH_SalVentAttn_TempOccPar_2  LH_SalVentAttn_Med_2  RH_DorsAttn_Post_5  RH_Cont_Par_1  LH_DorsAttn_Post_2  LH_SomMot_3  RH_DorsAttn_FEF_1  LH_DorsAttn_FEF_1  RH_SalVentAttn_FrOperIns_1  LH_SalVentAttn_FrOperIns_1  RH_SomMot_7 | 100  81  80  80  72  71  57  53  47  46  43  40  39  36  35 | RH_SalVentAttn_Med_2  RH_SalVentAttn_TempOccPar_2  LH_SalVentAttn_Med_3  RH_DorsAttn_Post_5  RH_DorsAttn_FEF_1  LH_SalVentAttn_ParOper_1  RH_Cont_Par_1  LH_SomMot_3  LH_SalVentAttn_Med_1  LH_DorsAttn_PrCv_1  RH_SomMot_7  RH_SalVentAttn_Med_1  LH_DorsAttn_Post_2  LH_DorsAttn_FEF_1  RH_DorsAttn_Post_2 | 100  96  84  80  80  75  66  60  56  52  50  48  47  46  43 |
| MC | RH_Default_Temp_1  RH_Limbic_TempPole_1  LH_Limbic_TempPole_1  LH_Vis_1  RH_Vis_1  RH_DorsAttn_Post_1  RH_Default_Temp_2  LH_Vis_2  RH_SomMot_1  RH_SalVentAttn_FrOperIns_1  RH_Vis_3  RH_Default_Temp_3  LH_Vis_3  LH_Default_Temp_1  RH_SomMot_3 | 100  90  88  87  84  82  78  74  71  65  63  63  60  57  55 | LH_Limbic_TempPole_1  RH_Vis_1  LH_Default_Temp_1  RH_Limbic_TempPole_1  LH_Vis_1  LH_Vis_3  LH_SalVentAttn_FrOperIns_1  RH_Vis_2  RH_Default_Temp_3  RH_Default_Temp_1  RH_Vis_3  RH_Default_Temp_2  LH_Default_Temp_2  LH_DorsAttn_Post_1  LH_SomMot_1 | 100  94  90  89  88  81  72  71  61  60  57  56  55  51  45 | LH_SomMot_1  LH_Default_Temp_1  LH_SomMot_3  LH_DorsAttn_Post_1  LH_Vis_3  RH_Vis_2  LH_Limbic_TempPole_1  LH_Default_Temp_2  LH_Vis_1  LH_Limbic_TempPole_2  RH_Vis_1  RH_SomMot_1  LH_SomMot_2  LH_SalVentAttn_FrOperIns_1  RH_Vis_3 | 100  97  95  93  90  89  88  86  86  85  83  67  66  60  60 |
| MP | RH_SalVentAttn_Med_3  LH_SalVentAttn_Med_2  LH_DorsAttn_Post_5  LH_DorsAttn_FEF_1  RH_SalVentAttn_FrOperIns_1  LH_SomMot_8  RH_DorsAttn_Post_5  LH_SalVentAttn_Med_1  RH_DorsAttn_FEF_1  LH_Default_PFCv_2  RH_SomMot_1  LH_SomMot_1  RH_SalVentAttn_Med_1  LH_Cont_PFCl_4  RH_SomMot_6 | 100  96  89  80  76  70  69  63  56  52  51  50  49  42  41 | RH_SalVentAttn_Med_3  LH_DorsAttn_Post_5  RH_DorsAttn_Post_5  LH_SalVentAttn_Med_2  LH_SalVentAttn_Med_1  RH_SomMot_6  LH_Cont_pCun_1  LH_DorsAttn_FEF_1  LH_SomMot_8  RH_SalVentAttn_Med_2  RH_DorsAttn_FEF_1  RH_Default_pCunPCC_2  LH_SomMot_7  LH_Default_pCunPCC_2  RH_SalVentAttn_FrOperIns_1 | 100  98  91  91  84  82  74  62  62  61  57  46  40  35  34 | RH_SalVentAttn_FrOperIns_1  LH_DorsAttn_Post_5  LH_SalVentAttn_Med_2  RH_SalVentAttn_Med_3  RH_SomMot_1  RH_DorsAttn_Post_5  LH_SomMot_1  LH_SomMot_8  RH_SomMot_4  LH_DorsAttn_FEF_1  RH_SomMot_3  LH_DorsAttn_Post_1  RH_SomMot_6  LH_Cont_pCun_1  RH_DorsAttn_FEF_1 | 100  86  83  81  78  68  60  59  55  39  38  36  35  30  30 |

Table S2. Schaefer parcellations (100 parcels, 7 networks)

| LH_Vis_1 | RH_Vis_1 |
| --- | --- |
| LH_Vis_2 | RH_Vis_2 |
| LH_Vis_3 | RH_Vis_3 |
| LH_Vis_4 | RH_Vis_4 |
| LH_Vis_5 | RH_Vis_5 |
| LH_Vis_6 | RH_Vis_6 |
| LH_Vis_7 | RH_Vis_7 |
| LH_Vis_8 | RH_Vis_8 |
| LH_Vis_9 | RH_SomMot_1 |
| LH_SomMot_1 | RH_SomMot_2 |
| LH_SomMot_2 | RH_SomMot_3 |
| LH_SomMot_3 | RH_SomMot_4 |
| LH_SomMot_4 | RH_SomMot_5 |
| LH_SomMot_5 | RH_SomMot_6 |
| LH_SomMot_6 | RH_SomMot_7 |
| LH_DorsAttn_Post_1 | RH_SomMot_8 |
| LH_DorsAttn_Post_2 | RH_DorsAttn_Post_1 |
| LH_DorsAttn_Post_3 | RH_DorsAttn_Post_2 |
| LH_DorsAttn_Post_4 | RH_DorsAttn_Post_3 |
| LH_DorsAttn_Post_5 | RH_DorsAttn_Post_4 |
| LH_DorsAttn_Post_6 | RH_DorsAttn_Post_5 |
| LH_DorsAttn_PrCv_1 | RH_DorsAttn_PrCv_1 |
| LH_DorsAttn_FEF_1 | RH_DorsAttn_FEF_1 |
| LH_SalVentAttn_ParOper_1 | RH_SalVentAttn_TempOccPar_1 |
| LH_SalVentAttn_FrOperIns_1 | RH_SalVentAttn_TempOccPar_2 |
| LH_SalVentAttn_FrOperIns_2 | RH_SalVentAttn_FrOperIns_1 |
| LH_SalVentAttn_PFCl_1 | RH_SalVentAttn_Med_1 |
| LH_SalVentAttn_Med_1 | RH_SalVentAttn_Med_2 |
| LH_SalVentAttn_Med_2 | RH_Limbic_OFC_1 |
| LH_SalVentAttn_Med_3 | RH_Limbic_TempPole_1 |
| LH_Limbic_OFC_1 | RH_Cont_Par_1 |
| LH_Limbic_TempPole_1 | RH_Cont_Par_2 |
| LH_Limbic_TempPole_2 | RH_Cont_PFCl_1 |
| LH_Cont_Par_1 | RH_Cont_PFCl_2 |
| LH_Cont_PFCl_1 | RH_Cont_PFCl_3 |
| LH_Cont_pCun_1 | RH_Cont_PFCl_4 |
| LH_Cont_Cing_1 | RH_Cont_Cing_1 |
| LH_Default_Temp_1 | RH_Cont_PFCmp_1 |
| LH_Default_Temp_2 | RH_Cont_pCun_1 |
| LH_Default_Par_1 | RH_Default_Par_1 |
| LH_Default_Par_2 | RH_Default_Temp_1 |
| LH_Default_PFC_1 | RH_Default_Temp_2 |
| LH_Default_PFC_2 | RH_Default_Temp_3 |
| LH_Default_PFC_3 | RH_Default_PFCv_1 |
| LH_Default_PFC_4 | RH_Default_PFCv_2 |
| LH_Default_PFC_5 | RH_Default_PFCdPFCm_1 |
| LH_Default_PFC_6 | RH_Default_PFCdPFCm_2 |
| LH_Default_PFC_7 | RH_Default_PFCdPFCm_3 |
| LH_Default_pCunPCC_1 | RH_Default_pCunPCC_1 |
| LH_Default_pCunPCC_2 | RH_Default_pCunPCC_2 |

Table S3. Abbreviations Schaefer atlas

| Vis | Visual network |
| --- | --- |
| SomMot | Somatomotor network |
| DorsAttn_Post | Dorsal attention network – posterior cortex |
| DorsAttn_PrCv | Dorsal attention network – precentral ventral frontal cortex |
| DorsAttn_FEF | Dorsal attention network – frontal eye fields |
| SalVentAttn_ParOper | Salience/ventral attention network – parietal operculum |
| SalVentAttn_FrOperIns | Salience/ventral attention network – frontal operculum insula |
| SalVentAttn_PFCl | Salience/ventral attention network – bilateral prefrontal cortex |
| SalVentAttn_Med | Salience/ventral attention network – medial lobe |
| Limbic_OFC | Limbic network – orbitofrontal cortex |
| Limbic_TempPole | Limbic network – temporal pole |
| Cont_Par | Control network – parietal lobe |
| Cont_PFCl | Control network – bilateral prefrontal cortex |
| Cont_pCun | Control network – precuneus cortex |
| Cont_Cing | Control network – cingulum |
| Default_Temp | Default mode network – temporal lobe |
| Default_Par | Default mode network – parietal lobe |
| Default_PFC | Default mode network – prefrontal cortex |
| Default_pCunPCC | Default mode network – precuneus posterior cingulate cortex |

*
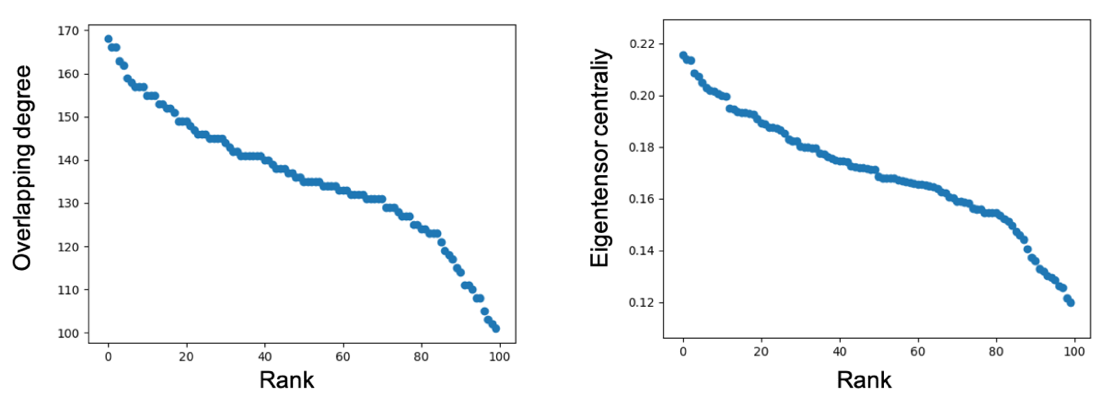
*

Figure S1. Rank distributions of overlapping degree and eigentensor centrality for the SC – FC – MC multiplex (at 20% connectivity density as an example). The rank distribution of both eigentensor centrality and overlapping degree shows an exponential behavior at low and at high ranks. Therefore, both distributions show a small fraction of nodes with a distinguishably higher/lower value than the majority of the nodes.
